# Tardive akathisia related to the anti-hypertensive agent Sevikar-a case report

**DOI:** 10.1186/s40360-017-0148-3

**Published:** 2017-06-05

**Authors:** Men-Ting Hsieh, Pao-Yen Lin, Chia-Jen Tsai, Chiung-Chih Chang, Yu Lee

**Affiliations:** 1 Department of Psychiatry, Kaohsiung Chang Gung Memorial Hospital, Chang Gung University College of Medicine, 123 Dapi Road, Niao-Sung District, Kaohsiung City, 833 Taiwan; 2 Department of Endocrinology, Kaohsiung Chang Gung Memorial Hospital, Chang Gung University College of Medicine, Kaohsiung, Taiwan; 3 Department of Neurology, Kaohsiung Chang Gung Memorial Hospital, Chang Gung University College of Medicine, Kaohsiung, Taiwan

**Keywords:** Sevikar, Anti-hypertensive agent, Tardive akathisia, Amlodipine, Calcium-channel blocker, Olmesartan, Angiotensin II receptor blocker

## Abstract

**Background:**

Tardive akathisia (TA) is a subtype of tardive syndrome, and its etiology is still uncertain. Sevikar is an anti-hypertensive agent containing both amlodipine and olmesartan, and has never been reported to have an adverse reaction in patients with tardive syndrome.

**Case presentation:**

A 57-year-old woman who took Sevikar for hypertension for 10 years developed TA one and a half years before receiving any psychiatric treatment. After switching from Sevikar to bisoprolol, she reported obvious improvement in her akathisia.

**Conclusions:**

It is noteworthy that her TA developed before receiving any antidepressant medication, and that her TA improved after discontinuation of Sevikar. In light of these pharmacodynamic properties, it is therefore concluded that use of amlodipine and olmesartan might have caused TA in this patient. We reported this rare case to remind clinicians to be aware of possible akathisia when using amlodipine and olmesartan in combination as anti-hypertensive agents.

## Background

Tardive akathisia (TA) is a subtype of tardive syndrome, an involuntary movement disorder that is mostly related to prolonged use of antipsychotics [1, 2]. The etiology of TA is still unclear. Sevikar is a complex anti-hypertensive agent containing both amlodipine and olmesartan. Amlodipine is a calcium-channel blocker (CCB) that blocks slow calcium channels in a dose-dependent pattern [3]. Olmesartan is an angiotensin II receptor blocker (ARB) [4]. Sevikar-related tardive syndrome has never been reported in the literature. Here we report a woman who took Sevikar for hypertension for 10 years and developed TA one and a half years before seeking help at psychiatric clinic.

## Case presentation

A 57-year-old married woman came to our psychiatric outpatient clinic due to depressed mood for one and a half years. Accompanying symptoms included insomnia, loss of interest, fatigue, memory impairment, decreased appetite, and suicidal ideation. Also, bilateral lower limbs restlessness had been noted by her daughter for one and a half years. This lower limbs restlessness was accompanied with frequent pacing. According to her daughter and herself, the lower limbs restlessness and pacing occurred before she started taking antidepressants. She initially sought help from a neurologist for the above problems one year prior to this visit and was diagnosed as having major depressive disorder. She was prescribed escitalopram 10 mg daily and clonazepam 0.75 mg daily for one year, with full compliance. Her depressed mood and other depressive symptoms improved, but her insomnia and bilateral lower limbs restlessness persisted. Also, her akathisia aggravated during the most recent five months. Neurological examination showed mild crossing and uncrossing of the legs while sitting. Her akathisia was rated 3 on the Extrapyramidal Symptoms Rating Scale (ESRS) [5]. We completed a survey including blood tests and images to rule out other underlying diseases that would possibly cause the symptoms. The blood tests included thyroid function, hemoglobin A1C (HbA1c), homocysteine, copper level, ceruloplasmin, rheumatologic factors (IgG, IgM, IgA, RF, ANA, ENA), cryoglobulin, RPR (rapid plasma reagin), hepatitis antigen/antibody (HBsAg and anti-HCV) and antiphospholipid antibody (anti-beta 2 GPI-IgG, cardiolipin-IgG, and cardiolipin-IgM). Laboratory data revealed elevation of HbA1c whose level was 8.4%. Otherwise, she had no thyroid dysfunction, homocysteinuria, Wilson’s disease, rheumatologic diseases, immunological diseases, syphilis, hepatitis or antiphospholipid syndrome. Brain CT showed a negative result. Medical history included hypertension, type 2 diabetes mellitus (DM), hyperlipidemia, and hyperthyroidism status post-thyroidectomy under treatment with a thyroxine supplement. She maintained Sevikar (amlodipine 5 mg + olmesartan medoxomil 20 mg) daily, atorvastatin 40 mg daily, and Glucomet (glyburide 5 mg + metformin 500 mg) twice daily for 10 years.

The patient was given escitalopram 10 mg daily, clonazepam 2 mg nightly, and zaleplon 10 mg nightly at our outpatient clinic. However, her akathisia did not improve much. Thus, we informed her endocrinologist about her akathisia, and suggested her antihypertensive agent be switched from Sevikar (amlodipine 5 mg + olmesartan medoxomil 20 mg) to bisoprolol 2.5 mg daily. During a 3-month follow-up, the patient reported subjective improvement in her akathisia, which was rated 1 on the ESRS.

## Discussion

It is noteworthy that the patient’s symptoms of TA developed before she received any antidepressant medication and improved after stopping Sevikar. Based on the temporal relationship, we can infer that her TA might have been caused by Sevikar, rather than an antidepressant agent.

In addition to Sevikar, the patient had also been taking atorvastatin, Glucomet, and thyroxine for more than 10 years. There is no available data in the current literature that suggests these medications might induce TA. Two case reports describe metformin-induced dystonia, but not akathisia [6, 7]. In addition, DM has been suggested to be related to dystonia [8, 9]; however, this was not supported by other reports [10, 11].

Aside from being induced by medication, akathisia may sometimes be caused by depression with anxiety. Although the patient’s akathisia appeared together with depression, her akathisia persisted and even aggravated after her depression had improved. Thus, it is less likely that her akathisia was directly caused by depression.

The etiology of TA is still unclear. One possible explanation is that an imbalance between dopaminergic/cholinergic and dopaminergic/serotonergic systems may result in TA [12]. Some researchers suggested that TA is caused by an imbalance between the core and the shell of the nucleus accumbens, partly due to overstimulation of the locus ceruleus [13]. Still, others hypothesized that TA may develop due to dopamine receptor hypersensitivity in the basal ganglia, which is related to postsynaptic dopamine blockade [14].

As mentioned previously, amlodipine is a CCB and olmesartan is an ARB, and both are effective for treating hypertension. CCBs can be categorized chemically into two groups, dihydropyridines and non-dihydropyridines. Amlodipine is categorized as a dihydropyridine; non-dihydropyridines include cinnarizine, flunarizine, verapamil, and others. In the current literature, most movement disorders induced by CCBs were reported to be due to the use of cinnarizine and flunarizine [15, 16]. A possible mechanism of CCB-induced movement disorders is thought to be the CCB’s property of dopamine D2 antagonism. Prolonged use of CCB might induce dopamine hypersensitivity in the nigrostriatal pathway and cause TA.

Research has shown that angiotensins may increase dopamine release in the nigrostriatal-dopaminergic pathway [17]. Thus, blocking angiotensin receptors may result in decreased dopamine release. However, data is even more limited when it comes to the relationship between ARBs and akathisia. It has been speculated that long-term inhibition of the nigrostriatal-dopaminergic system by ARB might increase the possibility of dopamine hypersensitivity and lead to TA. Based on the pharmacodynamic properties of these drugs, either amlodipine or olmesartan might have caused TA in this patient.

## Conclusions

This case, linking Sevikar, a complex drug containing both amlodipine and olmesartan, to the development of TA is important since there is limited data suggesting their association in the literature. To our knowledge, this is the first reported case of TA related to Sevikar. We report this rare case to remind clinicians to be cautious about this possible side effect, and that early discontinuation of Sevikar might improve the symptoms of TA.

## Abbreviations

ARB: Angiotensin II receptor blocker
CCB: Calcium-channel blocker
DM: Diabetes mellitus
ESRS: Extrapyramidal symptoms rating scale
HbA1c: Hemoglobin A1c
RPR: Rapid plasma regain
TA: Tardive akathisia
